# Combining Transcranial Direct Current Stimulation With Tai Chi to Improve Dual-Task Gait Performance in Older Adults With Mild Cognitive Impairment: A Randomized Controlled Trial

**DOI:** 10.3389/fnagi.2021.766649

**Published:** 2021-12-13

**Authors:** Ying-Yi Liao, Mu-N Liu, Han-Cheng Wang, Vincent Walsh, Chi Ieong Lau

**Affiliations:** ^1^Department of Gerontological Health Care, National Taipei University of Nursing and Health Sciences, Taipei, Taiwan; ^2^Department of Psychiatry, Taipei Veterans General Hospital, Taipei, Taiwan; ^3^Division of Psychiatry, School of Medicine, National Yang Ming Chiao Tung University, Taipei, Taiwan; ^4^Institute of Brain Science, National Yang Ming Chiao Tung University, Taipei, Taiwan; ^5^Memory and Aging Center, University of California, San Francisco, San Francisco, CA, United States; ^6^Department of Neurology, Shin Kong Wu Ho-Su Memorial Hospital, Taipei, Taiwan; ^7^College of Medicine, National Taiwan University, Taipei, Taiwan; ^8^Applied Cognitive Neuroscience Group, Institute of Cognitive Neuroscience, University College London, London, United Kingdom; ^9^Institute of Biophotonics, National Yang Ming Chiao Tung University, Taipei, Taiwan; ^10^College of Medicine, Fu-Jen Catholic University, New Taipei City, Taiwan; ^11^Department of Neurology, University Hospital, Taipai, Macao SAR, China

**Keywords:** mild cognitive impairment, tDCS, Tai Chi, dual-task gait, executive function

## Abstract

**Introduction:** Engaging in a secondary task while walking increases motor-cognitive interference and exacerbates fall risk in older adults with mild cognitive impairment (MCI). Previous studies have demonstrated that Tai Chi (TC) may improve cognitive function and dual-task gait performance. Intriguingly, with emerging studies also indicating the potential of transcranial direct current stimulation (tDCS) in enhancing such motor-cognitive performance, whether combining tDCS with TC might be superior to TC alone is still unclear. The purpose of this study was to investigate the effects of combining tDCS with TC on dual-task gait in patients with MCI.

**Materials and Methods:** Twenty patients with MCI were randomly assigned to receive either anodal or sham tDCS, both combined with TC, for 36 sessions over 12 weeks. Subjects received 40 min of TC training in each session. During the first 20 min, they simultaneously received either anodal or sham tDCS over the left dorsolateral prefrontal cortex. Outcome measures included dual-task gait performance and other cognitive functions.

**Results:** There were significant interaction effects between groups on the cognitive dual task walking. Compared to sham, the anodal tDCS group demonstrated a greater improvement on cadence and dual task cost of speed.

**Conclusion:** Combining tDCS with TC may offer additional benefits over TC alone in enhancing dual-task gait performance in patients with MCI.

**Clinical Trial Registration:** [www.ClinicalTrials.gov], identifier [TCTR20201201007].

## Background

Mild cognitive impairment (MCI) is an intermediate stage of cognitive decline between normal aging and dementia that carries a high risk of developing into dementia (Petersen, 2011). However, only limited therapeutic options are currently available, with no drugs approved for treating MCI (Petersen et al., 2018). Walking is a typical dual task that is an essential part of daily life. Examples of cognitive activities carried out while walking are talking, interpreting traffic lights, or planning daily activities. According to the capacity-sharing theory, when two attention-demanding tasks are performed at the same time in a condition of limited attentional resources, the performance of at least one of the tasks will deteriorate (Li et al., 2019). Individuals with cognitive impairment may be particularly susceptible to dual-task interference because there are fewer attentional resources available for the simultaneous performance of secondary tasks (Nascimbeni et al., 2015). Studies have reported that dual-task interference is exaggerated in elderly people with cognitive dysfunction (Yogev-Seligmann et al., 2008). Significantly decreased gait velocity and increased variability in stride time when shifting between a single task and a dual task have been previously reported in older adults with MCI (Muir et al., 2012). Differences in velocity, stride length, and stride time during single or dual tasks have also been well documented between patients with MCI and cognitively normal controls (Bahureksa et al., 2017). Due to a strong link between dual-task gait performance and cognitive impairment, dual-task interference or dual task cost (i.e., the difference between the scores of the dual-task and single-task performances) was shown to be predictive of future cognitive decline and greater risk of falls (De Cock et al., 2017).

The association between prefrontal cortex (PFC) activity and dual-task gait performance has been previously established (Nóbrega-Sousa et al., 2020). A functional near-infrared spectroscopy study showed that, comparing with control, MCI was associated with attenuated increases in hemodynamic response over the PFC in response to the greater cognitive demands imposed by dual task walking (Holtzer and Izzetoglu, 2020). Therefore, enhancing neural activation in the PFC may optimize dual-task gait performance. Recently, there has been increasing interest in non-pharmacological treatments for MCI (Wang et al., 2014). Among these, transcranial direct current stimulation (tDCS) provides non-invasive neuromodulation by delivering a low electrical current intensity (0.5–2.0 mA) through the scalp (Shin et al., 2015). An increasing number of studies support the efficacy of applying anodal tDCS over the dorsolateral prefrontal cortex (DLPFC) in enhancing cognitive performance (e.g., processing speed, working memory, and executive function) in patients with MCI (Meinzer et al., 2015; Gomes et al., 2019). More specifically, dual-task gait performance (e.g., stride time and variability) can be improved immediately after a single or 10 sessions of tDCS in elderly individuals (Manor et al., 2016, 2018). TDCS may augment cortical excitability and neuroplasticity in the same brain area stimulated by cognitive or motor practice (Bolognini et al., 2009). Previous studies have suggested that tDCS preferentially affects synapses already undergoing plasticity (Kronberg et al., 2017). A meta-analysis revealed that coupling tDCS with cognitive training appears to have a mild positive effect on memory and language in elderly individuals with MCI, but the results have been inconclusive (Cruz Gonzalez et al., 2018b). In addition to cognitive training, physical exercise has been shown to reduce cognitive decline in patients with MCI (Song et al., 2018). Since both tDCS and physical exercise have beneficial effects on cognitive function, combining the two may have synergic effects. To date, only one study has investigated the acute effects of this combination on dual-task walking performance (Schneider et al., 2021). The study showed that, compared to sham tDCS, tDCS delivered during motor-cognitive walking decreased the dual-task cost of walking speed in older adults. However, the repetitive effects of combining tDCS with physical exercise on dual-task gait performance have not been empirically addressed (Steinberg et al., 2018).

Tai Chi (TC) is a kind of mind-body exercise particularly beneficial to older adults with MCI because it involves movement recall, task switching, attention, and visuospatial processing simultaneous with physical movements (Li et al., 2014). Previous studies reported improvements in executive function, language, learning, and memory after TC training (Miller and Taylor-Piliae, 2014; Wayne et al., 2014). Since TC is a combination of cognitive and motor tasks, it may also improve single-task gait and dual-task gait performance, including gait initiation and dual-task variability (Vallabhajosula et al., 2014; Wayne et al., 2015). Applying tDCS concurrently with TC practice may enhance the beneficial effects of TC, although to date, no study has investigated their combinational effect. Therefore, the purpose of this study was to compare the effects of tDCS coupled with TC and TC alone on dual-task gait performance in patients with MCI. A battery of cognitive tasks assessing executive function was also included as secondary cognitive measures. We hypothesized that anodal tDCS coupled with TC would exert more beneficial effects on dual-task gait performance than sham tDCS coupled with TC.

## Materials and Methods

### Participants

All participants were recruited from the Dementia Center, Shin-Kong Wu Ho-Su.

Memorial Hospital, Taiwan. Sociodemographic variables such as age, sex, body weight, height, and body mass index, years of education and handedness were obtained from interviews and medical records. The inclusion criteria were as follows: (1) subjects aged 65 years and over; (2) a diagnosis of MCI according to Petersen’s criteria (Petersen, 2004); (3) a global rating of 0.5 on the Clinical Dementia Rating (CDR) scale; and (4) able to walk more than 10 m without walking aids. The exclusion criteria included (1) dementia; (2) brain tumors; (3) previous cerebral infarction or hemorrhage; (4) other known neurodegenerative or neuropsychiatric conditions; (5) the presence of an unstable orthopedic disease interfering with participation in the study; and (6) an education level of less than 6 years (elementary school).

### Study Design

This study was a double-blinded (assessor) randomized controlled trial. The participants were randomly assigned to either the anodal tDCS or sham tDCS group *via* a sealed envelope. The subjects participated in 40 min of TC training in each session. During the first 20 min of training, they simultaneously received either real or sham tDCS. Both groups attended sessions three times a week for 12 weeks for a total of 36 sessions. An experienced physical therapist conducted individual TC training, and all participants received the intervention individually. The assessor, who was always blinded to the group assignments, measured the outcomes at baseline and between 24 and 48 h (anodal tDCS group: 29.8 ± 8.3 h; sham group: 30.9 ± 8.1 h) after completing the 36 sessions. The gait assessment and cognitive assessment were independently performed with gait performance first followed by the cognitive assessments. The study was approved by the ethics committee of Shin-Kong Wu Ho-Su Memorial Hospital, and all subjects gave their informed consent prior to the beginning of the study.

### Transcranial Direct Current Stimulation

A battery-operated constant direct-current Stimulator Plus (NeuroConn, Ilmenau, Germany) was used in the present study. The current of the stimulation was administered at 2 mA *via* a saline-soaked pair of sponge electrodes with dimensions of 5 × 7 cm (35 cm^2^) to optimize the stimulation at the left DLPFC. The anode electrode was centered over F3 based on the 10–20 international system, whereas the cathode electrode was centered at the contralateral (right) supraorbital area, i.e., Fp4. For the anodal tDCS group, 2 mA of direct current was delivered for 20 min with a 10-s ramp-up period. For the sham condition, while the stimulation parameters and electrode montage remained the same as those for the anodal condition, the current was delivered only for the initial 30 s and then ramped down to 0 mA. This simulated the tingling sensation of active stimulation that was indistinguishable from anodal tDCS but with presumably negligible effects on the brain. All participants were tDCS-naïve subjects who, in both the tDCS and sham groups, were led to believe that they were receiving real tDCS. For the purpose of simultaneously delivering tDCS during TC, all subjects wore a semitransparent backpack that carried the tDCS device during practice. In all sessions, the impedance of all electrodes was monitored throughout the entire period of stimulation to maintain values under 5 kΩ.

### Concurrent Tai Chi Training and Transcranial Direct Current Stimulation

The participants were taught the 24 forms of Yang Style TC for 12 weeks. Yang Style TC is a popular and widely practiced form of TC characterized by gentle, broad, slow, and open movements, with an emphasis on breath and motor awareness. Forty minutes of TC included warming up, learning new forms, practicing learned forms, and cooling down. TC was taught and led by a certified coach, and the participants learned and practiced 2–3 new forms of the 24-form TC weekly. The BORG Perceived Exertion Scale after the TC session to control the exercise intensity was set at a score of 13–14 (somewhat hard) (Stuckenschneider et al., 2020). Both the anodal tDCS group (a-tDCS+TC) and the sham group (sham+TC) received online stimulation during the first 20 min of TC training by the methods described above.

## Outcome Measures

### Primary Outcome Measure (Gait Performance)

Gait parameters were measured by the Gait Up system (Gait Up, Lausanne, Switzerland), which is a wearable device with good validity and reliability (Mariani et al., 2010; Dadashi et al., 2013). Two wireless inertial sensors with the triaxial Gait Up accelerometers were placed on the dorsal side of the shoes. Three conditions were designed for assessing gait performance: (1) the single-task condition: walking at the subject’s preferred speed; (2) the cognitive dual-task condition: walking while executing a serial 3′s subtraction task that starts from a randomized 3-digit number (e.g., 100, 97, 94…) and (3) the motor dual-task condition: walking while carrying a tray with glasses of water. The participants were asked to walk three trials in each condition, while spatiotemporal parameters recorded during each trial, namely, speed (cm/s), stride length (cm), and cadence (step/min), were averaged from the three trials for the statistical analysis. Dual-task interference was quantified by the dual task cost, which was calculated with the following equation: [dual task cost speed (%) = dual-task walking speed – single-task walking speed]/single-task walking speed * 100% (Plummer and Eskes, 2015).

### Secondary Outcome Measures (Cognitive Task Performance)

#### The Montreal Cognitive Assessment

The MoCA has been a useful screening instrument for cognitive impairment, including that associated with MCI and Alzheimer’s disease (AD) (Nasreddine et al., 2005). The MoCA assesses seven subcategories of cognitive function: visual-spatial/executive, naming, memory, attention, language, abstraction, and orientation. The scores can range from 0 to 30, and higher scores indicate better global cognitive function. The reliability and validity of the Taiwanese version of the MoCA are 0.88 and 0.86, respectively (Tsai et al., 2012).

#### Visual Working Memory

Visual Working Memory (VWM) was assessed by a change detection task designed with E-prime 2.0 (Psychology Software Tools Inc.). The task was performed on a laptop with a 14-inch screen placed approximately 70 cm from the participant. Three different colored squares with no repetitive color in the same array were randomly displayed across both sides on a screen with a presentation duration of 100 ms, during which participants were asked to remember the colors. Following a 900-ms retention interval, another set of colored squares was presented until a response was given from the participant. While the locations and orientation of the squares were kept constant, subjects were instructed to identify as quickly as possible whether the presented memory array was identical in color (change/no change) to the previous array. Each subject performed 4 separate blocks of 30 trials in total, and performance in the change detection task was determined by reaction time as well as accuracy, estimated by d-prime (d′) *via* average hit rate and false-alarm rate.

#### Tower of London Task

The Tower of London (ToL) task is a classic neuropsychological test for the assessment of planning and problem solving (Shallice, 1982). Subjects must move an array of colored beads mounted on three vertical rods to match a particular goal arrangement. For each participant, increasingly complex goals are set such that the participant has to complete three tasks, the first requiring two moves to reach the goal, the second requiring four moves, and the third requiring five moves. Total time and accuracy were analyzed. A computerized version of the ToL task was used [the Psychology Experiment Building Language (PEBL)].^1^

#### Trail Making Test

The Trail Making Test (TMT)-A and the Chinese version of the TMT-B examine visual attention and task switching, which are components of executive function. The TMT-A consisted of 25 circles numbered (1–25) randomly distributed on a sheet of paper. The test required the participants to connect the 25 consecutive numbers in ascending order as quickly as possible. The Chinese version of the TMT-B involved 12 circles numbered (1–12) and 12 Chinese characters representing the 12 animal signs of the Chinese zodiac. The participants were required to draw connecting lines between the 12 consecutive numbers in ascending order with zodiac signs inserted alternately between numbers (i.e., 1–rat–2–ox–3–tiger, etc.). The less time spent completing the tests indicated better performance. The time to complete each test was recorded.

#### Stroop Color and Word Test

The current study used the incongruous subtask of the Chinese version of the Stroop Color and Word Test (SCWT). In this task, color words (i.e., black, blue, red, yellow) were printed in an inconsistent color ink with the character (i.e., the word blue printed in red ink). The participants had to name the color of the ink rather than state the word/character as quickly as they could and within a limited time. The number of correct answers in 45 s (SCWT number) and time to name 45 characters (SCWT time) were our outcomes.

#### Chinese Version of the Verbal Learning Test

The Chinese Version of the Verbal Learning Test (CVVLT) is a validated short version of the original California Verbal Learning Test that aims to measure episodic memory in elderly Chinese-speaking populations (Chang et al., 2010). The examiner read the nine two-character nouns on the list aloud at 1-s intervals in a fixed order over four learning trials. The participants were encouraged to recall as many nouns as possible after each trial. The total number of nouns correctly recalled during these four trials as well as 10 min later were recorded.

### Data Analysis

Sociodemographic, neuropsychological and gait data analyses were performed using SPSS 20.0 software (SPSS Inc., Chicago, IL, United States). Descriptive statistics were generated for all variables, and variable distributions are expressed as the means ± standard deviations or as numbers. Intergroup differences in baseline characteristics were analyzed using independent *t*-tests or chi-square tests. Two-way ANOVAs with repeated measures were used to determine the effects of TC on cognitive function assessed by neuropsychological tasks and gait parameters assessed by Gait Up. We assessed the interaction between Intervention Group (a-tDCS + TC, sham + TC) and Assessment Time (baseline, post-intervention). Significance was set at *p* < 0.05. For the similar tasks involving executive function (TOL, TMT, SCWT), we performed Bonferroni corrections due to multiple comparisons. The significance was set at *p* < 0.017.

## Results

**FIGURE 1:**
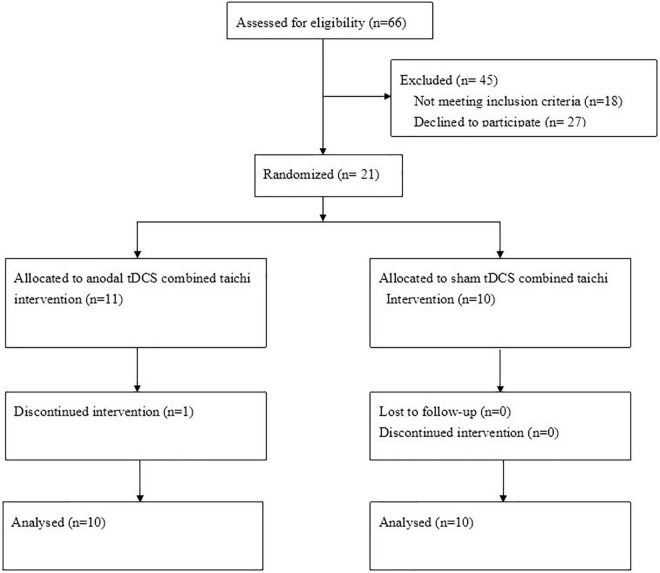
Flow chart of the study design.

As shown in the flowchart (Figure 1), 21 qualified patients were randomly assigned to either the a-tDCS+ TC group or the sham + TC group. Of the 21 participants, one patient in the a-tDCS+ TC group did not complete the study due to a low adherence to the protocol (4/36 sessions), the data of this participant was thus withdrawn from the analysis. As such, a total of 20 participants completed all the interventions and assessments. None of the participants reported any adverse events. A brief postintervention interview showed that participants in both groups believed that they had received genuine tDCS. The demographic characteristics of the patients are shown in Table 1.

**TABLE 1:** Baseline demographic characteristics of patients (*n* = 20).

	a-tDCS + TC group (*n* = 10)	sham + TC group (*n* = 10)	*p*-value
Age, years	72.6 ± 4.1	73.1 ± 4.6	0.801
Sex, female/male	8/2	5/5	0.129
Dominant hand, right/left	10/0	10/0	1
Education	8.5 ± 3.6	12.1 ± 2.9	0.026
Height, cm	156.8 ± 7.6	157.7 ± 7.8	0.799
Body weight, kg	53.7 ± 10.2	58.1 ± 9.0	0.324
Body mass index, kg/m^2^	21.8 ± 1.8	23.1 ± 2.1	0.143

*Data are presented as means ± SDs or numbers.*

### Dual-Task Gait Performance

**FIGURE 2:**
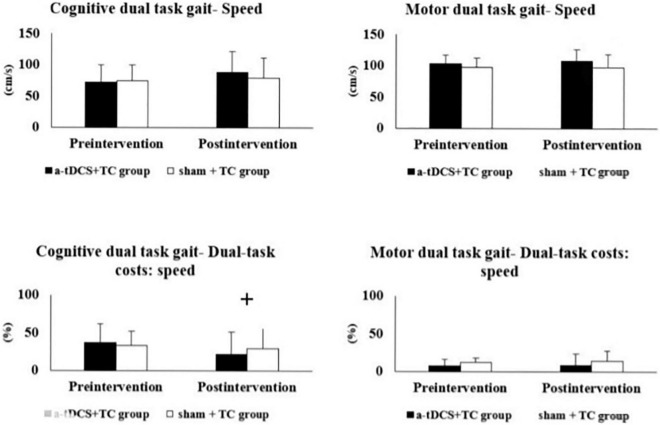
Comparisons of single- and dual-task gait performance in the a-tDCS+TC group and sham+TC group. ^+^*p* < 0.05 significant interactions between group and time.

The results of the single- and dual-task performance are shown in Table 2 and Figure 2. In regard to cognitive dual-task performance, we observed significant interaction effects across both cadence [*p* = 0.006*, *F*_(1, 18)_ = 9.743, η^2^ = 0.351] and speed outcomes [dual task cost of speed, *p* = 0.041*, *F*_(1, 18)_ = 4.837, η^2^ = 0.212]. In the a-tDCS+TC group, *post hoc* tests showed significant improvements after training in speed (*p* = 0.048), cadence (0.003) and dual task cost of speed (0.002) of the cognitive dual-task gait.

**TABLE 2:** Comparisons of dual-task gait performance of the a-tDCS+TC group and sham+TC group.

	a-tDCS + TC group (*n* = 10)		sham + TC group (*n* = 10)		Time * Group
	Pre	Post	Pre	Post	
**Single-task gait**					
Speed (cm/s)	111.7 ± .11.1	116.6 ± .13.0	113.2 ± 17.5	114.8 ± .19.8	0.443, *F*_(1, 18)_ = 0.614, η^2^ = 0.033
Stride length (cm/s)	116.4 ± 12.30	121.5 ± 14.1	122.8 ± .18.3	122.7 ± .19.3	0.156, *F*_(1, 18)_ = 2.197, η^2^ = 0.109
Cadence (step/minute)	114.4 ± 8.96	114.6 ± 10.1	110.0 ± .9.7	111.4 ± .8.3	0.659, *F*_(1, 18)_ = 0.201, η^2^ = 0.011
**Cognitive dual-task gait**					
Speed (cm/s)	72.9 ± 26.9	87.9 ± 25.4	74.1 ± .34.1	79.5 ± .32.4	0.187, *F*_(1, 18)_ = 1.881, η^2^ = 0.095
Dual-task costs: speed (%)	37.3 ± 24.2	22.0 ± 19.36*	33.2 ± 29.1	29.7 ± 28.4	0.041*, *F*_(1, 18)_ = 4.837, η^2^ = 0.212
Stride length (cm/s)	109.4 ± 25.7	114.6 ± 22.5	106.2 ± .26.7	109.8 ± 24.0	0.748, *F*_(1, 18)_ = 0.106, η^2^ = 0.006
Cadence (step/minute)	74.3 ± 16.1	94.6 ± 9.8*	80.9 ± .31.6	83.5 ± 27.4	0.006*, *F*_(1, 18)_ = 9.743, η^2^ = 0.351
**Motor dual-task gait**					
Speed (cm/s)	102.9 ± 14.1	108.1 ± 15.0	97.4 ± 18.1	98.0 ± 20.7	0.463, *F*_(1, 18)_ = 0.563, η^2^ = 0.030
Dual-task costs: speed (%)	7.5 ± 9.1	8.5 ± 5.4	12.8 ± 15.8	13.9 ± 13.8	0.984, *F*_(1, 18)_ = 0.000, η^2^ = 0.000
Stride length (cm/s)	110.0 ± 15.4	112.4 ± 14.0	109.6 ± 15.7	114.3 ± 29.0	0.727, *F*_(1, 18)_ = 0.125, η^2^ = 0.007
Cadence (step/minute)	111.6 ± 10.9	112.8 ± 10.1	106.1 ± 11.1	100.7 ± 12.4	0.239, *F*_(1, 18)_ = 1.484, η^2^ = 0.076

Data are presented as means ± SDs or numbers. *p < 0.05.

### Cognitive Task Performance

The cognitive task performance before and after the intervention is illustrated in Table 3. There was some evidence of an interaction effect in the TMT-B [*p* = 0.018, *F*_(1, 18)_ = 6.706, η^2^ = 0.271], however this was not significant when adjusting for multiple comparisons (α = 0.017).

**TABLE 3:** Comparisons of cognitive task performance between the a-tDCS+TC group and sham+TC group.

	a-tDCS+TC group (*n* = 10)		Sham+TC group (*n* = 10)		Time × Group effect
	Pre-intervention	Post-intervention	Pre-intervention	Post-intervention	*p*-value
MoCA (score)	23.3 ± 4.63	24.9 ± 3.62	24.2 ± 3.45	25.3 ± 3.19	0.626, *F*_(1, 18)_ = 0.246, η^2^ = 0.014
**CCVLT (number)**					
Verbal memory	23.5 ± 4.47	26.0 ± 5.14	23.6 ± 3.16	26.3 ± 4.73	0.878, *F*_(1, 18)_ = 0.024, η^2^ = 0.001
Delayed recall	4.00 ± 2.86	6.50 ± 2.54	4.20 ± 2.39	5.40 ± 2.83	0.224, *F*_(1, 18)_ = 1.583, η^2^ = 0.081
VWM (d′)	1.36 ± 1.44	1.24 ± 1.00	0.90 ± 0.66	1.18 ± 0.78	0.321, *F*_(1, 18)_ = 1.046, η^2^ = 0.058
SCWT (number)	28.4 ± 5.94	32.2 ± 5.34	28.9 ± 8.47	32.4 ± 9.53	0.884, *F*_(1, 18)_ = 0.022, η^2^ = 0.001
SCWT (time)	74.1 ± 22.3	67.3 ± 11.7	79.0 ± 25.7	74.2 ± 18.5	0.784, *F*_(1, 18)_ = 0.077, η^2^ = 0.004
TMT-A (seconds)	47.5 ± 11.36	44.8 ± 8.99	49.8 ± 15.3	51.8 ± 17.0	0.326, *F*_(1, 18)_ = 1.022, η^2^ = 0.054
TMT-B (seconds)	120.6 ± 39.2	88.3 ± 35.6	110.8 ± 39.6	95.9 ± 30.4	0.018, *F*_(1, 18)_ = 6.706, η^2^ = 0.271
**ToL**					
Total time (seconds)	797.22. ± 571.33	672.65 ± 607.45	774.45 ± 547.85	587.19 ± 378.50	0.691, *F*_(1, 18)_ = 0.164, η^2^ = 0.009
Accuracy	6.20 ± 2.93	8.80 ± 2.04	7.20 ± 1.96	7.90 ± 2.18	0.068, *F*_(1, 18)_ = 3.756, η^2^ = 0.173

*Data are presented as means ± SDs or numbers.*

## Discussion

To the best of our knowledge, this is the first study to compare the combined effects of tDCS and TC vs. TC alone on dual-task gait performance and cognitive functions in patients with MCI. The main finding of our study is that 36 sessions of concurrent anodal tDCS and TC training was superior to sham stimulation with TC training in improving cognitive dual-task walking. Hence, our findings provide preliminary support for the effectiveness of combined tDCS and TC in improving aspects of cognitive-motor performance in this population.

### Evidence of Additive Effects of Transcranial Direct Current Stimulation and Tai Chi on Dual-Task Gait

Only a few studies have examined the enhancing effects of tDCS on dual-task performance in healthy adults (Zhou et al., 2014; Wrightson et al., 2015; Ljubisavljevic et al., 2019; Schneider et al., 2021) and patients with Parkinson’s disease (Schabrun et al., 2016). Most of these studies have explored the immediate effects after stimulation (offline) (Zhou et al., 2014; Wrightson et al., 2015; Manor et al., 2016, 2018; Schneider et al., 2021), with one study evaluating the effects during the application of tDCS (online) (Ljubisavljevic et al., 2019). Unlike these studies, which focused on the immediate effects of a single tDCS session, our study applied multisession tDCS and demonstrated the effects of coupling tDCS and TC 24–48 h beyond the entire period of intervention. Previous studies have shown that a combination of tDCS and aerobic exercise might provide acute synergic effects on cognition (Steinberg et al., 2018), our study expanded this type of investigation to gait and their long-lasting effects. Nevertheless, our study design cannot answer the question of whether multisession a-tDCS coupled with TC would exert additional effects compared to a-tDCS alone. According to another study, tDCS targeting the left DLPFC immediately mitigated dual-task costs to walking (Zhou et al., 2021). Although our experimental design did not include a “tDCS only” group, we may speculate that multisession a-tDCS alone could exert a similar beneficial effect on dual-task gait performance.

The strength of our study is that our subjects were able to receive tDCS and TC concurrently. Recent studies have shown that tDCS tends to modulate the same brain networks that are already activated by a task or brain event, suggesting that the impact of tDCS on cognitive or motor tasks is state-dependent (Stagg et al., 2011; Martin et al., 2014). Thus, coupling tDCS and TC together could potentially enhance the same TC-related brain networks that are targeted by tDCS and create a synergistic effect in cortical excitability and neuroplasticity (Martin et al., 2014). Another strength of the current study is that the augmenting effects of combining TC and tDCS were transferred to other unlearned tasks involving similar cognitive domain, i.e., cognitive dual-task gait. This suggests that the beneficial effects of combining TC and tDCS could potentially be transferred and applied to other daily activities in patients with MCI and thus improve their quality of life.

The enhancing effects of TC on dual-task gait performance (gait speed and stride time variability) has been reported in previous studies (Wayne et al., 2015; Li et al., 2019), yet their training effects required as long as 6 months to achieve. In comparison, our relatively shorter period of TC practice, i.e., 3 months, might not have been sufficient to yield a significant impact on gait performance. As such, we were unable to see substantial dual-task gait improvements in the sham group. Interestingly, significant intergroup improvements in the a-tDCS+TC group may be attributed to the facilitating effect of tDCS. A recent meta-analysis showed that tDCS was able to induce considerable improvement in balance control and dual-task gait conditions in older adults in a short time period (from 1 to 16 sessions) (Guo et al., 2020). Our more extended period of intervention, i.e., 36 sessions in 3 months, may enhance the facilitating effects of tDCS and led to more robust improvements than TC alone.

### Proposed Mechanisms of Transcranial Direct Current Stimulation-Augmented Tai Chi Effects on Dual-Task Performance

We chose the left prefrontal cortex as our tDCS target because it has been implicated in the allocation of cognitive resources between two concurrently performed tasks (Collette et al., 2005), notably during dual-task gait (Doi et al., 2013; Al-Yahya et al., 2016). As such, we hypothesized that the tDCS online effect over the augmented neural circuit of the prefrontal cortex under TC practice might improve cognitive capacity and reduce the attention needed to perform the cognitive task, thereby permitting greater attention to be shifted toward performing a concurrent task (e.g., walking). The bottleneck theory of dual-task interference suggests that when two tasks are performed simultaneously, one task involving the same neural network will be postponed until the other prioritized task is completed (Ruthruff et al., 2001). Based on this theory, tDCS-related improvements in our study may have resulted from improved processing speed and shortened time delay between the cognitive and walking tasks (Redfern et al., 2002).

Walking while performing serial subtraction in older adults with MCI is a highly demanding task that requires alternating momentary processing capacity and filtering out all signals that are irrelevant to the task (Montero-Odasso et al., 2009). We speculate that without the augmenting effect of tDCS, the improvements in cognitive function in the sham group may not have been substantial enough to transfer to improvements in dual task gait. A higher intensity, duration, and challenge might be required in future TC studies.

### Effects of Transcranial Direct Current Stimulation and Tai Chi on Other Tasks Involving Executive Function

There was some evidence of an interaction effect in the TMT-B, however, this was not significant when adjusting for multiple comparisons. The TMT-B and SCWT can be seen as similar tasks associated with cognitive flexibility and working memory (Scarpina and Tagini, 2017). Arguably, the augmenting effects of tDCS and TC may not have consistently transferred to all tasks we tested for executive function. While the mechanisms leading to selective enhancement of individual tasks engaging the same cognitive domain remain unclear, the positive aftereffects of tDCS on performance tended to be more prominent if the task closely resembled the task performed during tDCS application (Stagg et al., 2011; Martin et al., 2014). Since TC is a dual task resembling cognitive dual task gait, the latter was subject to the greatest aftereffect of tDCS. Interestingly, the TMT-B shares some similarities with dual task gait in that both tasks assess divided attention—the ability to simultaneously attend to two different stimuli and respond to the multiple demands of the surroundings. Indeed, a recent study showed that poor TMT performance was directly associated with altered dual task prioritization in elderly individuals (Hobert et al., 2011). The acute effects of tDCS on enhancing divided attention assessed *via* the TMT has been demonstrated in patients with Parkinson’s disease and MCI (Cruz Gonzalez et al., 2018a; Bueno et al., 2019). Our finding showing a favorable trend of tDCS relative to sham in TMT performance warrants future studies on a larger cohort of MCI subjects.

### Limitations and Future Directions

Limitations of this study include the lack of a “no intervention” group and a “tDCS only” group, which may further clarify the mechanisms underlying our results. Second, trials with larger samples and longer periods of follow-up are needed to confirm the prolonged effects of tDCS+TC on cognition and dual task gait performance. Third, the non-significant training effects of the motor dual task gait in both groups might be due to the relatively simple motor task (carrying a glass of water). Our finding supports a previous study that gait performance in patients with MCI was more susceptible to cognitive than motor secondary task (Hunter et al., 2018). Future studies may involve more complicated motor tasks to assess motor complexity influences on gait performance. Fourth, although we adopted a traditional tDCS montage with the anodal electrode over the left DLPFC and the reference electrode over the contralateral supraorbital region, we were unable to demonstrate the precise distribution and dosage of electric current in specific brain regions. Future studies should incorporate brain imaging and computational models to determine the accurate location and dosage of current, as well as changes in brain activities following this novel combination of non-pharmacological interventions for MCI. Lastly, the large number of statistical tests and the small sample size may have increased Type I errors based on the multiple comparisons in gait and cognitive outcome measurements.

## Conclusion

To our knowledge, this is the first study to examine the clinical effects of combining tDCS and TC in patients with MCI. Our results suggest that tDCS may boost the positive effects of TC on gait, which may in turn transfer to other unlearned tasks in patients with MCI. We conclude that coupling tDCS with TC may be an effective non-pharmacological intervention for patients with MCI.

## Data Availability Statement

The raw data supporting the conclusions of this article will be made available by the authors, without undue reservation.

## Ethics Statement

The studies involving human participants were reviewed by the Institutional Human Research Ethics Committee of Shin-Kong Wu Ho-Su Memorial Hospital, and the study protocol was registered at http://www.clinicaltrials.in.th/ (TCTR20201201007 on 1 December, 2020). The patients/participants provided their written informed consent to participate in this study.

## Author Contributions

Y-YL: conceptualization, methodology, funding acquisition, data collection and analysis, resources, and writing—original draft and editing. M-NL and H-CW: data collection and analysis and resources. VW: supervision and writing—review and editing. CL: conceptualization, methodology, funding acquisition, data collection and analysis, visualization, writing—original draft, and review and editing. All authors read and approved the final manuscript.

## Conflict of Interest

The authors declare that the research was conducted in the absence of any commercial or financial relationships that could be construed as a potential conflict of interest.

## Publisher’s Note

All claims expressed in this article are solely those of the authors and do not necessarily represent those of their affiliated organizations, or those of the publisher, the editors and the reviewers. Any product that may be evaluated in this article, or claim that may be made by its manufacturer, is not guaranteed or endorsed by the publisher.

## Footnotes

1
http://pebl.sourceforge.net/

## Abbreviations

AD: Alzheimer’s disease
CDR: Clinical Dementia Rating
CVVLT: Chinese version of the Verbal Learning Test
DLPFC: dorsolateral prefrontal cortex
MCI: mild cognitive impairment
MoCA: Montreal Cognitive Assessment
PEBL: Psychology Experiment Building Language
PFC: prefrontal cortex
SCWT: Stroop color and word test
tDCS: transcranial direct current stimulation
TC: Tai Chi
TMT: Trail Making Test
ToL: Tower of London
VWM: visual working memory.
